# Heme iron intake and risk of new-onset diabetes in a Mediterranean population at high risk of cardiovascular disease: an observational cohort analysis

**DOI:** 10.1186/1471-2458-13-1042

**Published:** 2013-11-04

**Authors:** Jose Candido Fernandez-Cao, Victoria Arija, Nuria Aranda, Monica Bullo, Josep Basora, Miguel Angel Martínez-González, Javier Díez-Espino, Jordi Salas-Salvadó

**Affiliations:** 1 Unidad Nutrición y Salud Pública, Universitat Rovira i Virgili Reus, Tarragona, Spain; 2 Unidad de Soporte a la Investigación Tarragona-Reus, Instituto Universitario de Investigación en Atención Primaria Jordi Gol (IDIAP Jordi Gol), Tarragona, Spain; 3 Institut d’Investigació Sanitària Pere Virgili, Universitat Rovira i Virgili, Tarragona, Spain; 4 Unitat de Nutrició Humana, Universitat Rovira i Virgili Reus, Tarragona, Spain; 5 CIBERobn Fisiopatología de la Obesidad y Nutrición, Instituto de Salud Carlos III, Madrid, Spain; 6 Department of Preventive Medicine and Public Health, University of Navarra, Madrid, Spain; 7 Centro de Salud de Tafalla, Servicio Navarro de Salud-Osasunbidea, Tafalla, Spain

## Abstract

**Background:**

Several epidemiological studies have observed an increased risk of type 2 diabetes mellitus (T2DM) among subjects with a higher consumption of red and processed meat. Heme iron intake has been directly associated with a higher risk of T2DM in healthy adult Chinese and U.S populations. The objective of the present study was to evaluate the association between heme iron intake and the incidence of T2DM in a Mediterranean population at high cardiovascular risk.

**Methods:**

We assessed a subset of participants in the PREDIMED trial as an observational cohort, followed up for a maximum of eight years. We initially included 1073 non-diabetic subjects (57.1% women) aged 67.3 ± 6.0 years, at high cardiovascular risk. Diet was assessed at the study baseline using a validated, semi-quantitative food frequency questionnaire.

**Results:**

During the follow-up period 131 diabetics were newly diagnosed. The risk of developing T2DM was assessed using baseline heme iron intake and proportional hazard models, first unadjusted, then adjusted for energy, and finally adjusted for dietary, anthropometric, socio-demographic and lifestyle variables. Significant direct associations with the incidence of T2DM were found for heme iron (Hazard Ratio [HR] 1.30, 95% confidence interval [CI], 1.02 to 1.66). Secondarily, we have also observed that coffee (HR:0.93, 95% CI, 0.89 to 0.98) and alcoholic beverages (HR: 1.02, 95% CI, 1.01 to 1.04) were also found to reduce and increase the risk of T2DM, respectively.

**Conclusion:**

High dietary intake of heme iron was associated with an increased risk of developing T2DM in a Mediterranean population at high cardiovascular risk.

**Trial registration:**

Identifier: ISRCTN35739639.

## Background

In recent years the prevalence of T2DM has increased worldwide. It has been estimated that the number of people aged 20 to 79 years affected by T2DM will increase from 285 million in 2010 (6.4%) to 439 million in 2030 (7.7%) [1].

Diet is one of the components of lifestyle that has most been studied in the prevention of T2DM. A recent meta-analysis reported that red meat, and particularly processed meat [2], is associated with a greater risk of T2DM incidence. In recent years, some studies in healthy adult Chinese [3,4] and U.S populations [5-7] suggest that the component of meat responsible for this association could be the intake of total iron [3] or heme iron [4-7].

The deregulation of iron homeostasis has been implicated in the origin of such pathologies as cancer, Alzheimer’s disease, cardiovascular disease and T2DM, among others [8]. Although the exact mechanism by which iron induces T2DM is not clear, it has been speculated that, being a pro-oxidant element, it strengthens the formation of hydroxyl radicals [9], thus favouring situations of oxidative stress and contributing to insulin resistance or long-term pancreatic failure [9].

The association between heme iron intake and increased risk of T2DM has been studied in the general population, but not in individuals at high cardiovascular risk. Therefore, the objective of the present study was to evaluate the effect of heme iron intake on the incidence of T2DM in a nondiabetic Mediterranean population at high cardiovascular risk.

## Methods

The study design was an observational cohort analysis of a subset of non-diabetic participants in the PREDIMED trial [10], who were followed up between 1 and 8 years (median 4.85 ± 1.28 years). PREDIMED is a large multicentre, randomized, controlled trial that attempts to test the efficacy of the Mediterranean diet (MeDiet) in the primary prevention of cardiovascular disease. Two traditional Mediterranean diets were evaluated – one enriched with extra virgin olive oil and the other with mixed nuts – along with a control low-fat diet normally recommended for cardiovascular disease prevention in a high-risk population. Full details of the PREDIMED trial (http://www.controlled-trials.com/ISRCTN35739639) have been published elsewhere [10].

### Study participants

The present study was conducted on 1 073 subjects: 460 men aged 55 to 80 years, and 613 women aged 60 to 80 years from two PREDIMED trial centers (Reus and Pamplona). The subjects included were non-diabetics and had three or more of the following cardiovascular risk factors: current smoker (>1 cigarette/day for the past month), high blood pressure (systolic ≥ 140 mmHg or diastolic ≥ 90 mmHg or taking blood pressure medication), LDL cholesterol ≥ 169 mg/dl or lipid-lowering treatments, HDL cholesterol ≤ 40 mg/dL in men or ≤ 50 mg/dL in women; body mass index (BMI) ≥ 25 kg/m^2^; and family history of premature coronary disease (≤ 55 years in men and ≤ 60 years in women). Exclusion criteria were a previous history of cardiovascular disease (coronary heart disease, stroke or peripheral arterial disease); any severe chronic illness; alcohol or drug abuse; BMI > 40 kg/m^2^; more than a 5% loss in body weight in the last year; and a history of allergy to nuts [10]. The Clinical Research Ethics Committee of the Hospital Sant Joan de Reus approved the study protocol and all participants provided written informed consent.

During the follow-up a total of 131 incident cases of T2DM were diagnosed using the American Diabetes Association criteria [11] (fasting blood glucose ≥ 7.0 mmol/L or blood glucose ≥ 11.1 mmol/L after an oral dose of 75 g glucose, measured annually). A second test with the same criteria was required to confirm the diagnosis. Incident T2DM cases were confirmed by the PREDIMED study’s Clinical Events Committee.

### Measurements

Dietary intake was determined at baseline using a validated 137-item, semi-quantitative food frequency questionnaire [12]. For the present analysis, food was grouped according to its nutritional composition. Finally, consumption of the following food groups was calculated: dairy; meat, fish and eggs; cereals, legumes and nuts; vegetables; fruits; oils and fats; candies and sweetened beverages; alcoholic beverages; tea and coffee. Total energy and nutrient intake were calculated on the basis of Spanish food composition tables [13]. Sociodemographic, anthropometric (weight, height, waist circumference) and biochemical data were also recorded at baseline and on each annual visit to identify health problems such as obesity (BMI ≥ 30 kg/m^2^), hypercholesterolemia (total cholesterol ≥ 240 mg/dl), hypertriglyceridemia (triglycerides ≥ 200 mg/dl) and hypertension (systolic blood pressure ≥ 140 mmHg and/or diastolic blood pressure ≥ 90 mmHg). The last three cardiovascular risk factors were defined according to the Third Report of the National Cholesterol Education Program (NCEP) Expert Panel on Detection, Evaluation, and Treatment of High Blood Cholesterol in Adults (Adult Treatment Panel III) [14].

The participants were divided into three grups: 0 – control group; 1 – Mediterranean Diet supplemented with olive oil; and 2 – Mediterranean Diet supplemented with nuts. Subjects were assessed as smokers or non-smokers. Education was recoded into two categories: high-medium and low. Finally, we created a categorical variable of heme iron intake: 1 – low (< 3.173 mg), 2 – medium (3.173–4.130 mg) and 3 – high (> 4.130 mg) consumers.

### Statistical analysis

The data are presented as a percentage or average ± standard deviation. Statistical testing included chi-square tests for qualitative variables and the t-Test for quantitative data.

First, we studied whether there was a linear relationship between heme iron intake and T2DM. Because the association between heme iron intake and T2DM was linear and at the border of significance (P = 0.05), we decided to analyze the association in the total population with several proportional hazard models. The first model used raw data with unadjusted heme iron intake. The second model used data adjusted for energy intake. And the final model was adjusted for dietary, anthropometric, socio-demographic and lifestyle variables: for example, age, sex, waist circumference, smoking, education level and intervention group. Some components of foods that can interfere with heme iron absorption were also adjusted for: for example, fruit, meat, fish and eggs [animal protein], cereals, legumes and nuts [vegetable protein], calcium, alcohol beverages, tea and coffee (the last two representing the consumption of polyphenols). Fruit was included as an adjustment variable because consumption was significantly higher in the group of non-diabetics. The variable “time to onset of T2DM” was measured in days. No interactions were found between sex and age.

Because the association between heme iron intake and T2DM was linear and at the border of significance, we decided to analyze the association for each tertile of heme iron intake, using the same adjustment variables and the last proportional hazard model.

Significance was set at P < 0.05. The data were analysed using version 20.0 of the SPSS statistical package for Windows.

## Results

A total of 12.2% of the initial sample developed T2DM with a median follow-up of 4.8 ± 1.3 years. Table 1 shows the general baseline characteristics of non-incident and incident diabetics. At baseline, weight, BMI, waist circumference and smoking prevalence were significantly higher in incident than in non-incident T2DM subjects.

**Table 1:** **Baseline general characteristics of participants in incident and non**-**incident diabetics**

**VARIABLES**	**TOTAL n = 1073**		
	**NON INCIDENT** **n = 942 (87.8%)**	**INCIDENT DIABETICS** **n = 131 (12.2%)**	**p**
Age (years)	67.3 ± 6.0	66.3 ± 5.7	0.073
Weight (kilograms)	75.3 ± 10.8	79.2 ± 10.6	< 0.001
IMC (kg/m2)	29.4 ± 3.1	30.4 ± 3.1	0.001
Waist circumference (cm)	97.2 ± 9.9	101.8 ± 9.3	< 0.001
Women (%)	57.7	52.7	0.271
Obesity (%)	41.5	48.1	0.153
Hypercholesterolemia (%)	35.8	35.7	0.992
Hypertriglyceridemia (%)	16.3	23.8	0.239
Hypertension (%)	65.6	62.8	0.719
Smoking (%)	16.3	25.2	0.012
Medium-high education (%)	26.9	27.9	0.807

Results expressed as mean ± standard deviation or percentages.

Table 2 shows the average consumption of the different food groups, the total energy intake and the consumption of main nutrients at baseline in non-incident and incident diabetics in total subjects and subjects classified according to tertiles of heme intake. The group of incident T2DM subjects consumed less fruit (*P* = 0.010) and coffee (*P* = 0.001), and more heme iron (*P* = 0.036) than the non-incident group in the total sample but not by tertiles. Only in the second (*P* = 0.040) and third (*P* = 0.029) tertiles was coffee consumption significantly higher in non-incident than incident diabetics.

**Table 2:** **Baseline food**, **energy and nutrient consumption in incident cases of diabetes and controls**

**VARIABLES**	**TOTAL n = 1073**		**p**	**FIRST TERTILE**		**SECOND TERTILE**		**THIRD TERTILE**	
	**NON INCIDENT** **n = 942 (87.8%)**	**INCIDENT DIABETICS** **n = 131 (12.2%)**		**NON INCIDENT** **n = 324 (90.8%)**	**INCIDENT DIABETICS** **n = 33 (9.2%)**	**NON INCIDENT** **n = 310 (86.6%)**	**INCIDENT DIABETICS** **n = 48 (13.4%)**	**NON INCIDENT** **n = 358 (86.0%)**	**INCIDENT DIABETICS** **n = 50 (14.0%)**
Heme iron (mg/day)	3.7 ± 1.19	3.9 ± 1.3	0.036	2.5 ± 0.3	2.6 ± 0,4	3.6 ± 0.3	3.7 ± 0.3	5.1 ± 0.9	5.1 ± 1.2
Non-heme iron (mg/day)	12.0 ± 3.23	11.8 ± 3.4	0.538	11.1 ± 3.0	10.3 ± 2.7	11.9 ± 2.8	12.0 ± 3.6	13.2 ± 3.5	12.8 ± 3.4
Iron (mg/day)	15.7 ± 3.80	15.8 ± 4.0	0.886	13.6 ± 3.1	12.8 ± 2.8	15.5 ± 2.9	15.7 ± 3.6	18.2 ± 3.9	17.9 ± 3.8
Vitamin C (mg/day)	170.5 ± 69.56	165.0 ± 58.1	0.394	153.5 ± 62.9	140.6 ± 64.9	175.2 ± 68.9	161.6 ± 53.2	183.6 ± 73.4	184.5 ± 52.0
Calcium (mg/day)	978.6 ± 328.73	963.5 ± 368.6	0.628	853.7 ± 289.7	820.4 ± 246.6	997.5 ± 309.9	927.6 ± 313.9	1091.0 ± 341.7	1092.4 ± 440.6
Fiber (g/day)	23.0 ± 6.74	22.2 ± 6.7	0.189	21.9 ± 6.6	20.5 ± 7.3	22.8 ± 5.9	21.9 ± 6.1	24.4 ± 7.4	23.6 ± 6.8
Carbohydrates (g/day)	241.1 ± 72.28	228.3 ± 79.8	0.062	222.5 ± 70.5	188.2 ± 47.9	238.2 ± 63.5	233.3 ± 79.8	263.4 ± 76.5	250.0 ± 87.7
Protein (g/day)	90.1 ± 19.76	90.8 ± 19.5	0.687	75.0 ± 14.0	73.3 ± 9.9	89.2 ± 12.4	89.77 ± 13.6	106.9 ± 17.7	103.4 ± 20.1
Total fat (g/day)	102.7 ± 27.02	104.5 ± 28.1	0.482	89.5 ± 22.3	89.0 ± 26.4	102.3 ± 22.1	103.8 ± 27.0	117.4 ± 28.7	115.4 ± 25.8
Total energy (Kcal/day)	2 328.5 ± 553.55	2 322.9 ± 607.0	0.916	2059.3 ± 484.7	1912 ± 362.9	2305.5 ± 446.8	2344.6 ± 610.3	2634.8 ± 76.5	2573.4 ± 596.9
Dairy (g/day)	369.6 ± 212.0	351.1 ± 233.2	0.357	342.5 ± 201.8	344.2 ± 184.1	382.5 ± 209.8	335.1 ± 200.4	385.1 ± 222.4	371.1 ± 288.1
Meat, fish and eggs (g/day)	231.4 ± 67.1	237.7 ± 60.4	0.303	177.2 ± 41.1	181.4 ± 37.7	229.1 ± 41.9	238.0 ± 40.3	290.6 ± 60.3	237.7 ± 60.4
Cereals, legumes & nuts (g/day)	196.5 ± 91.2	196.1 ± 101.0	0.961	185.3 ± 90.7	164.9 ± 60.2	188.6 ± 82.2	199.5 ± 109.1	216.4 ± 97.1	213.5 ± 110.9
Vegetables (g/day)	279.6 ± 108.8	276.3 ± 105.1	0.741	254.3 ± 96.6	243.6 ±105.8	277.9 ± 103.5	250.0 ± 97.3	308.2 ± 119.1	323.2 ± 96.3
Fruits (g/day)	352.9 ± 181.3	315.4 ± 150.8	0.010	328.8 ± 170.6	278.8 ± 177.2	373.1 ± 186.1	329.5 ± 135.8	357.8 ± 185.0	326.0 ± 144.8
Oils & fats (g/day)	57.9 ± 22.0	60.5 ± 25.0	0.220	53.0 ± 19.9	52.5 ± 19.3	57.5 ± 21.0	62.4 ± 29.9	63.6 ± 23.8	63.9 ± 22.4
Alcoholic beverages (cc/day)	128.5 ± 190.9	17.0 ± 287.4	0.087	106.5 ± 185.2	91.1 ± 151.2	132.1 ± 210.3	174.4 ± 221.3	147.9 ± 173.7	225.7 ± 387.2
Coffee (cc/day)	31.5 ± 47.5	20.5 ± 32.9	0.001	27.2 ± 46.1	16.0 ± 33.4	31.4 ± 43.8	20.6 ± 31.5	36.0 ± 52.2	23.4 ± 34.3
Tea (cc/day)	5.3 ± 23,1	4.5 ± 13.2	0.720	4.6 ± 25.6	3.0 ± 10.9	5.8 ± 24.3	5.1 ± 14.1	5.4 ± 23.1	4.9 ± 13.2

Results expressed as mean ± standard deviation.

To evaluate the association between heme iron intake and various components of the diet with the risk of T2DM, proportional hazard models were applied (Table 3). The first model used raw data with unadjusted heme iron intake (HR = 1.13, 95% CI, 0.99 to 1.30; p = 0.075). The second model used data adjusted for energy intake (HR = 1.20, 95%CI, 1.02 to 1.42; p = 0.029). And the final model was adjusted for dietary, anthropometric, socio-demographic and lifestyle variables and showed that greater heme iron intake (HR = 1.30, 95% CI, 1.02 to 1.66; p = 0.037) was associated with an increased risk of T2DM onset. Although it was not our principal aim, we also observed an association between others components of the diet and the risk of T2DM. Thus, the consumption of alcohol beverages was associated with an increased risk of T2DM (HR = 1.02, 95% CI, 1.01 to 1.04), while coffee was associated with better protection against T2DM incidence (HR = 0.93, 95% CI, 0.89 to 0.98).

**Table 3:** Cox regression evaluating the association between heme iron intake and the risk of new onset diabetes

**VARIABLES**	**P**^**1**^	**HAZARD RATIO**	**95% Confidence interval for hazard ratio**	
			**Lower**	**Higher**
Model 1: Heme iron	0.075	1.13	0.99	1.30
Model 2: Heme iron +	0.029	1.20	1.02	1.42
Energy	0.210	0.98	0.94	1.01
Model 3: Heme iron +	0.037	1.30	1.02	1.66
Energy +	0.113	0.95	0.88	1.01
Animal protein +	0.155	0.97	0.93	1.01
Vegetable protein +	0.320	1.02	0.99	1.05
Fruit +	0.339	0.99	0.98	1.01
Alcoholic beverages +	0.005	1.02	1.01	1.04
Coffee +	0.005	0.93	0.89	0.98
Tea +	0.665	0.98	0.89	1.08
Calcium	0.747	1.01	0.95	1.08

Last model adjusted also for age (years), gender, waist circumference (cm) (*P* < 0.001; Exp(B) = 1.05), education level (medium-high, low), intervention group (control, olive oil and nuts) and smoking.

Heme iron (mg/day), energy intake (100 kcal/day), animal protein [meat, fish and eggs] (10 g/day), vegetables protein [cereals, legumes and nuts] (10 g/day), fruit (10 g/day), alcoholic beverages (20 cc/day), coffee (10 cc/day), tea (10 cc/day), calcium (100 mg/day).

^1^P-value for hazard ratio.

Furthermore, when we constructed the last proportional hazard model, by dividing subjects into tertiles of heme iron intake, we observed no significant association in either the first tertile of heme iron intake (HR = 1.58, 95% CI, 0.52 to 4.78; P = 0.175) or the second (HR = 1.59, 95% CI, 0.50 to 5.06; P = 0.013), but there was a significant association in the third tertile and T2DM (HR = 1.57, 95% CI, 1.09 to 2.27; P = 0.003).

## Discussion

Several epidemiological studies have linked excess iron deposits and risk of T2DM [3,4,15]. However, few studies have analysed the association between heme iron intake and T2DM [3-7,16]. The present prospective study shows that higher amounts of dietary heme iron are associated with a higher risk of T2DM. This is the first time that this association has been assessed in a large elderly Mediterranean population at high cardiovascular risk.

This association has been evaluated in four long-term prospective studies [5-7,16] and two cross-sectional studies [3,4]. However, most of them were conducted in healthy cohorts of U.S adults (Professionals Follow-up Study, Women’s Health Study, Nurse’s Health Study and MESA), and showed that the intake of heme iron is associated with an increase in the risk of T2DM [5-7]. Only one study found no association [16]. In the Health Professionals Follow-up Study cohort, heme iron derived from red meat consumption, but not total iron intake, was positively associated with risk of T2DM [5]. In the Iowa Women’s Health Study cohort of postmenopausal women, a high intake of heme iron and/or iron supplements was associated with increased incidence of T2DM, especially in subjects who consumed alcohol [7]. Lastly, in the Nurses Health Study, the association between T2DM incidence and iron intake was demonstrated only for heme iron intake, but not for total iron, iron supplements, or non-heme iron intake [6]. The association between total iron [3] or heme iron intake [4] and prevalent T2DM was also cross-sectionally observed in healthy adult Chinese populations. Finally, a recent meta-analysis and systematic review suggest that increased heme iron intake is associated with higher risk of T2DM [17]. The association between heme iron intake before pregnancy and/or during the early period of pregnancy and gestational diabetes was also recently reported in the Nurses Health Study II [18] and the U.S. Omega cohort [19].

These findings have been reinforced by the fact that several studies have found an association between elevated levels of iron and the risk of T2DM [3,4,15]. Recently, a study on the Potsdam EPIC cohort found that high ferritin levels – above 110 ng/mL in females and 280 ng/mL in males – were associated with an increased risk of T2DM [15].

Blood donations and phlebotomy have also been observed to have a positive effect on insulin sensitivity and risk of T2DM because they reduce body iron levels. Fernandez-Real et al. observed a decrease in body iron levels and improved insulin sensitivity in healthy subjects who made frequent blood donations (at least 2 in the previous 5 years) [20]. They also found that the number of donations was positively correlated with insulin sensitivity. The same authors had previously observed that both insulin secretion and sensitivity, and glycosylated hemoglobin levels improved in diabetic patients undergoing phlebotomy [21]. These results appear to show that iron reduction has a beneficial effect on the occurrence of T2DM and controls the parameters associated with T2DM. Interventional studies should be carried out to determine whether reducing the heme iron intake diminishes the risk of healthy subjects developing T2DM and improves control and management of patients with T2DM.

Although the exact mechanism by which iron induces T2DM and other diseases is unclear [8], there is increasing evidence to suggest that iron status has a considerable influence on the oxidative damage suffered by biomolecules such as DNA [22]. It has been speculated that this phenomenon can affect the physiopathology of such key events in the onset of T2DM as insulin resistance or deficiency [9]. For example, storage of iron in pancreatic β cells could affect β cell secretion [23], inducing apoptosis mediated by oxidative stress [24].

The bioavailability of heme iron is determined by diet and is probably one of the important factors that explains why heme iron is the component of the diet that has consistently been associated with a risk of T2DM by our study and others. For this reason, the following factors were included in the proportional hazard models: meat, fish and eggs [animal protein]; cereals, legumes and nuts [vegetable protein]; alcoholic beverages; tea and coffee and calcium. This last factor, in conjunction with polyphenols [25], inhibits heme iron absorption [26]. The effects of polyphenols were regarded as being indirectly due to the consumption of tea and coffee, which are rich in phenolic compounds. Alcohol beverages were included because it has been suggested that acute or chronic exposure to alcohol can suppress hepcidin expression in the liver, which increases the intestinal transport of iron into plasma [27]. In our study, the effect of vegetable protein, another new factor that influences heme iron absorption [28], was estimated from the consumption of cereals, legumes and nuts. Another important factor involved in heme iron absorption is animal protein [28]. In the proportional hazard models this is presented as “meats, fish and eggs [animal protein]”. Although the variable “meats, fish and eggs [animal protein]” shows collinearity with “heme iron”, we decided to include both in the proportional hazard models because they assess different aspects. “Heme iron” is associated with the quantity of iron in the diet and “meats and fish [animal protein]” with facilitating iron absorption. In addition, “meats, fish and eggs [animal protein]” improved the β coefficient of the primary variable, “heme iron”, and the general model, both of which support the inclusion of these variables.

The results of our study are in agreement with those of other epidemiological studies [3-7]: a high intake of heme iron seems to be associated with an increased risk of T2DM, also in a Mediterranean population at high cardiovascular risk. This relationship was particularly evident in our study at high doses of heme iron intake. Although it is not fully understood why results vary according to the form of iron intake, heme iron is more bioavailable because its absorption is independent of body iron status and elevated heme iron intake has been associated with high levels of serum ferritin [29], which in turn is related to greater risk of T2DM [4].

On the final proportional hazard model applied, we also observed a positive association between the consumption of alcohol beverages and an increased risk of T2DM. On the other hand, coffee consumption was associated with a reduced risk, as has previously been observed in other studies [30]. The negative relationship between coffee consumption and the risk of T2DM could also be explained by the positive relationship between caffeine and insulin sensitivity, and between decaffeinated coffee and β cell function [31]. Polyphenols, including coffee and tea polyphenols, seem to inhibit the uptake of intestinal hemo iron [25], thus reducing its adverse effects. In the case of alcohol beverages, the presence of alcohol seems to have a detrimental effect on the metabolism of iron. In American postmenopausal women aged 55 to 69 years, an association was observed between higher heme iron or supplemented iron intake and greater risk of developing T2DM, especially in those who also consumed alcohol [7]. High or moderate consumption of alcohol was associated with a significant increase in the risk of iron overload [32]. In this regard, Whitfeld et al. observed that ferritin and iron serum were increased by alcohol consumption [33], and this, in turn, was associated with a risk of TDM2 [34].

Because of the observational nature of our study, we cannot completely establish a cause-effect relationship between heme iron intake and the risk of developing T2DM. However, the covariates in the statistical models minimize the major sources of confounding. One of the strengths of the study was that the diagnosis of T2DM was not self-reported and was verified by a second analytical test, thus making the identification of new incident cases more reliable and accurate. However, some participants did not undergo an OGTT, so T2DM could only be diagnosed by fasting blood sugar ≥ 7.0 mmol/L confirmed by a second test. This might have falsely lowered overall incidence rates. Finally, the dietetic variables used in the analysis were obtained from a semiquantitative food frequency questionnaire that had been validated in the same population so that the possibility of error was reduced [12].

## Conclusion

In conclusion, the results of the present study show that an elevated heme iron intake was associated with a significant increase in the risk of T2DM incidence in a Mediterranean population at high cardiovascular risk.

## Competing interests

Dr. Jordi Salas-Salvadó is a non-paid member of the Scientific Advisory Board of the International Nut Council. The other authors have no conflict of interest affecting the conduct or reporting of the work submitted.

## Authors’ contributions

JCF performed the literature searches, contributed to the interpretation of the results and wrote the manuscript. VA supervised the statistical analyses and helped to interpret the results and revise the manuscript. NA and MB contributed to the interpretation of the results and revised the manuscript. JB coordinated the fieldwork and revised the manuscript. MAG conceived the study, participated in its design and revised the manuscript. JD revised the manuscript. JS conceived the study, participated in its design and revised the manuscript. All authors read and approved the final manuscript.

## Pre-publication history

The pre-publication history for this paper can be accessed here:

http://www.biomedcentral.com/1471-2458/13/1042/prepub
